# Do functional status and Medicare claims data improve the predictive accuracy of an electronic health record mortality index? Findings from a national Veterans Affairs cohort

**DOI:** 10.1186/s12877-022-03126-z

**Published:** 2022-05-18

**Authors:** William James Deardorff, Bocheng Jing, Sun Y. Jeon, W. John Boscardin, Alexandra K. Lee, Kathy Z. Fung, Sei J. Lee

**Affiliations:** 1 Division of Geriatrics, University of California, San Francisco, 490 Illinois Street, Floor 08, San Francisco, CA 94158 USA; 2 Geriatrics, Palliative and Extended Care Service Line, San Francisco Veterans Affairs Health Care System, San Francisco, CA USA

**Keywords:** Functional status, Physical function, Medicare data, Mortality prediction model

## Abstract

**Background:**

Electronic health record (EHR) prediction models may be easier to use in busy clinical settings since EHR data can be auto-populated into models. This study assessed whether adding functional status and/or Medicare claims data (which are often not available in EHRs) improves the accuracy of a previously developed Veterans Affairs (VA) EHR-based mortality index.

**Methods:**

This was a retrospective cohort study of veterans aged 75 years and older enrolled in VA primary care clinics followed from January 2014 to April 2020 (*n* = 62,014). We randomly split participants into development (*n* = 49,612) and validation (*n* = 12,402) cohorts. The primary outcome was all-cause mortality. We performed logistic regression with backward stepwise selection to develop a 100-predictor *base* model using 854 EHR candidate variables, including demographics, laboratory values, medications, healthcare utilization, diagnosis codes, and vitals. We incorporated functional measures in a *base + function* model by adding activities of daily living (range 0-5) and instrumental activities of daily living (range 0-7) scores. Medicare data, including healthcare utilization (e.g., emergency department visits, hospitalizations) and diagnosis codes, were incorporated in a *base + Medicare* model. A *base + function + Medicare* model included all data elements. We assessed model performance with the c-statistic, reclassification metrics, fraction of new information provided, and calibration plots.

**Results:**

In the overall cohort, mean age was 82.6 years and 98.6% were male. At the end of follow-up, 30,263 participants (48.8%) had died. The *base* model c-statistic was 0.809 (95% CI 0.805-0.812) in the development cohort and 0.804 (95% CI 0.796-0.812) in the validation cohort. Validation cohort c-statistics for the *base + function*, *base + Medicare*, and *base + function + Medicare* models were 0.809 (95% CI 0.801-0.816), 0.811 (95% CI 0.803-0.818), and 0.814 (95% CI 0.807-0.822), respectively. Adding functional status and Medicare data resulted in similarly small improvements among other model performance measures. All models showed excellent calibration.

**Conclusions:**

Incorporation of functional status and Medicare data into a VA EHR-based mortality index led to small but likely clinically insignificant improvements in model performance.

**Supplementary Information:**

The online version contains supplementary material available at 10.1186/s12877-022-03126-z.

## Introduction

Accurate prediction of an individual’s life expectancy can inform many clinical decisions, including cancer screening, medication management for chronic conditions, and advance care planning [1, 2]. While many mortality indices have been developed among community-dwelling older adults, they are often underused for several possible reasons: time burden associated with obtaining necessary variables and inputting them into a calculator, lack of knowledge about their availability on sites such as ePrognosis, and discomfort among clinicians in discussing prognostic estimates with patients, families, and caregivers [3–5]. The increasing availability of electronic health record (EHR) data allows for the creation of automated prediction models that rely on the vast number of clinical data elements in the EHR [6]. Thus, EHR-based life expectancy calculators may facilitate the uptake of prediction models by eliminating the need to manually input data.

One notable EHR-based mortality prediction model developed within the Veterans Affairs (VA) healthcare system is the Care Assessment Need (CAN) score, which provides risk estimates for hospitalization, death, and either hospitalization or death at 90 days and 1 year [7]. The original goal of this score was to identify high-risk patients enrolled in VA primary care clinics to improve care coordination and better target resources. Given that the focus of the CAN score is on 1-year mortality risk, it is unclear if the CAN score is applicable to clinical decisions such as cancer screening which require life expectancy estimates at longer time horizons. Therefore, we recently developed a life expectancy calculator using EHR data in adults 50 years and older at VA primary care clinics that was specifically designed to provide individualized longer-term life expectancy predictions to help guide screening and prevention decisions [8]. The final model included 93 predictors across a variety of domains, including demographics, diseases, medications, labs, vital signs, and healthcare utilization. The model performed comparably to other long-term mortality risk tools with an integrated area under the receiver operating characteristic curve (iAUC) value of 0.816 and good calibration across 1 to 10 years of mortality prediction.

However, one limitation of this life expectancy calculator is that information on functional status, such as dependencies in activities of daily living (ADLs) and instrumental activities of daily living (IADLs), was not included. Functional impairments have been repeatedly shown to improve mortality predictions in non-EHR based mortality indices involving community-dwelling older adults in part because they represent the end-impact of several chronic diseases [9–11]. However, this information is often not easily extractable or universally collected in EHR data, making it challenging to incorporate into EHR-based mortality prediction models. In 2009, the VA Office of Geriatrics and Extended Care encouraged clinics to assess functional status during primary care appointments in patients aged 75 years and older through questions regarding assistance with ADLs and IADLs, resulting in many VA medical centers routinely collecting ADL/IADL data [12]. Thus, we sought to determine whether incorporating functional status into an EHR-based mortality index improves prediction accuracy.

In addition to functional status, Medicare claims data is a second potential data source outside the VA EHR which may improve prediction model accuracy. While the VA health care system is the largest integrated health care system in the United States, many veterans supplement their healthcare through Medicare services outside the VA [13, 14]. Adding Medicare data to a VA EHR-based prediction model requires additional effort to obtain and link the data elements. To help guide future researchers on how much improvement can be expected by incorporating Medicare data to EHR data, we compared the predictive accuracy of EHR-based prediction models with and without Medicare data.

To determine whether the improvements in prediction accuracy with the addition of functional measures and Medicare data justify the additional data collection and data linkage burdens, we sought to compare model performance measures across four EHR-based mortality prediction models developed in veterans aged 75 years and older: 1) a *base* model utilizing only VA EHR data, 2) a *base + function* model incorporating EHR + functional data, 3) a *base + Medicare* model incorporating EHR + Medicare data, and 4) *base + function + Medicare* model incorporating EHR + functional+Medicare data.

## Methods

### Study population

Our study population included a nationally representative sample of veterans aged 75 years and older enrolled in VA primary care clinics in 2014 (*n* = 62,014). We restricted our population to 49 VA medical centers that collected data on functional measures. We defined an index visit for individuals in our cohort as the first primary care visit between January 1, 2014 and December 31, 2014. We used a 1-year “look-back” period from the date of the index visit in 2014 to assess six domains of predictor variables: demographics, disease diagnoses, medication use, laboratory results, vital signs, and healthcare utilization. We incorporated a split-sample design in which 80% of the full cohort were randomly sampled for model development (*n* = 49,612) and the remaining 20% for model validation (*n* = 12,402).

Our primary outcome was all-cause mortality over the enter follow-up period. Follow-up data on mortality was collected using the Veterans Health Administration’s Vital Status File through April 2020 as this was the latest date that we had information on an individual’s date of death. This method of mortality ascertainment has been shown to have high accuracy in previous studies [15, 16]. Each participant had between 5 years and 4 months (December 31, 2014 - April 30, 2020) and 6 years and 4 months (January 1, 2014 - April 30, 2020) of potential follow-up time over the study period. The median and mean years of potential follow-up time were 5.98 and 5.94 years, respectively.

### Candidate variables

We obtained 854 predictors from the EHR for the *base* model, including 2 demographic variables (age/gender), 271 disease diagnosis codes from inpatient and outpatient VA data files classified using the Healthcare Cost and Utilization Project (HCUP) Clinical Classifications Software (CCS), 365 medication classes classified as any use in the past year, 88 laboratory tests, and 9 vital signs [17]. For EHR utilization data, we created 119 types of healthcare visits, such as emergency department (ED) visits, hospitalizations, and various types of outpatient appointments, categorized as 0, 1 or > 1 visits. For missing data, we performed single stochastic mean imputation using a regression equation with all variables with any missingness included. Variables with missingness were pulse (*n* = 561; 0.9%), temperature (*n* = 3852; 6.2%), respiration (*n* = 3251; 5.2%), weight range (*n* = 17,811; 28.7%), weight change (*n* = 17,941; 28.9%), systolic blood pressure (*n* = 545; 0.9%), and body mass index (*n* = 25,330; 40.8%). Additional details regarding these EHR candidate predictors are provided in the Supplementary Methods (Additional File 1) and the previously published life expectancy calculator [8].

### Functional variables

At the VA sites that assessed functional status, clinical staff were prompted via dialog boxes to ask yes or no questions about dependencies in ADLs and IADLs. An example question might be, “Is the person able to take a bath/shower/sponge bath without the assistance of another person?” with answers dichotomized as “Yes, able to bathe independently” or “No, not able to bathe independently.” Five questions were asked related to ADLs, including bathing, eating, toileting, dressing, and transferring. Seven questions were asked related to IADLs, including managing finances, managing medications, going shopping, using the telephone, preparing food, doing housekeeping, and doing laundry. ADL scores (range 0-5) and IADL scores (range 0-7) were calculated with higher scores indicating a greater number of dependencies (ADLs) or needing help (IADLs). The answers were compiled into the Corporate Data Warehouse Health Factors Domain Dataset.

### Medicare data variables

We examined the inpatient, outpatient, and carrier Medicare files to obtain Medicare utilization data and diagnosis codes generated when veterans obtained services outside of the VA. Medicare data was obtained through the VA Information Resource Center’s VA/Centers for Medicare and Medicaid Services (CMS) Data for Research Project. The six Medicare utilization data variables included ED visits, hospitalizations, outpatient visits, length of stay (LOS) at a skilled nursing facility (SNF), duration in days of home health services, and number of durable medical equipment (DME) received in the year prior to the index date. The variables corresponding to number of ED visits, hospitalizations, and outpatient visits in the year prior to the index date were categorized as 0, 1, or > 1 visit. LOS at a SNF was categorized as either 0 for no days spent at a SNF, 1 for LOS between 1 and 10 days, 2 for LOS between 11 and 20 days, 3 for LOS between 21 and 30 days, and 4 for LOS greater than 30 days. Duration in days of home health (HH) services was categorized as 0 for no HH visits, 1 for 1-20 days, 2 for 21-40 days, 3 for 41-60 days, and 4 for greater than 60 days. The number of DME received within the one-year period was categorized as 0 for no DME received, 1 for 1 item received, 2 for 2-3 items received, 3 for 4-8 items received, and 4 for greater than 8 items received. Medicare diagnosis codes were classified using the HCUP CCS and combined with VA EHR diagnosis codes. Most Medicare diagnosis codes included in the predictor pool overlapped with the VA disease diagnosis codes. When compared to the VA diagnosis codes, there were six unique Medicare diagnosis codes (female infertility, abruptio placenta, polyhydramnios, respiratory distress syndrome, hemolytic jaundice, and drowning/submersion) that were additionally added although likely not relevant in our population.

### Statistical analysis

We first examined baseline characteristics in the development and validation cohorts. To build a *base* model using the development cohort (*n* = 49,612), we performed multivariable logistic regression with backward stepwise selection using a *p*-value greater than 0.001 for removal of variables. We chose not to use machine learning models based on previous work using VA EHR data and because in data settings such as ours with ample sample size (“large N, small p”), studies suggest traditional regression methods are equivalent to more opaque machine learning methods [18–20]. To facilitate comparison across the four models, we forced the backward stepwise selection procedure to include 100 final variables in each model. We chose to create a 100 predictor model due to results from our previously published life expectancy calculator using VA EHR data, where our least absolute shrinkage and selection operator (LASSO) Cox proportional hazards regression with a Bayesian information criterion (BIC)-optimized lambda suggested that a 100 predictor model appropriately balanced model bias and variance [8].

The *base* model only considered VA EHR predictors, and we did not include any functional measures or Medicare data variables. We repeated the variable selection process in the development cohort to create three additional 100-predictor models: *base + function* model (which included EHR variables and ADL/IADL scores as candidate predictors), *base + Medicare* model (which included EHR and Medicare utilization variables and diagnosis codes as candidate predictors), and *base + function + Medicare* model (which included EHR variables, ADL/IADL scores, and all Medicare data variables as candidate predictors).

We assessed the incremental value of additional predictors (functional status and Medicare data) to the *base* model in several ways. We compared the concordance statistic (c-statistic) across the four models, which assesses a model’s ability to separate individuals who did and did not have the event of interest. To assess for overfitting, we re-calculated the c-statistic in our validation cohort. We calculated reclassification measures such as the net reclassification improvement (NRI) and integrated discrimination improvement (IDI) [21, 22]. NRI measures the degree to which the additional measures were able to appropriately reclassify individuals who did and did not die. We pre-specified a two-category index with cut-off at 0.50 as we felt that this would represent a meaningful threshold for thinking about average life expectancy. The IDI reflects the difference in discrimination slopes between the base model and the model including the additional measures. It serves to quantify the value of added measures by calculating improvements in sensitivity and specificity integrated over all possible cut-offs. We also calculated the fraction of new information provided, which refers to the proportion of variation explained by additional predictors when added to the *base* model [23]. Calibration, which refers to the agreement between observed outcomes and predictions, was assessed visually by plotting the predicted probability of mortality (x-axis) by the observed proportion of mortality (y-axis) [24]. All statistical analyses were conducted using SAS version 9.4 (SAS Institute, Inc) and R version 4.03 (R Project for Statistical Computing). Additional details regarding the statistical analysis are provided in the Supplementary methods (Additional File 1).

## Results

Baseline characteristics were similar in the development (*n* = 49,612) and validation (*n* = 12,402) cohorts (Table 1). The mean age was 82.6 years and 98.6% were male. There was a high prevalence of common medical conditions such as hypertension (76.5%) and diabetes (35.8%). The median number of ADL and IADL dependencies was 0 with 92.9 and 70.2% of patients reporting no ADL and IADL dependencies in the development cohort, respectively. The distribution of ADL and IADL scores is shown in Supplementary Table S1. By the end of follow-up in April 2020, 30,263 participants (48.8%) had died.

**Table 1:** Selected baseline characteristics in the development and validation cohorts

Characteristic	Development cohort (***n*** = 49,612)	Validation cohort (***n*** = 12,402)
Demographics		
Age in years, mean (SD)	82.6 (4.8)	82.5 (4.8)
Male	48,918 (98.6%)	12,204 (98.4%)
Vital Signs		
BMI ≥30	9278 (18.7%)	2332 (18.8%)
SBP ≥140 mmHg	14,877 (30.0%)	3793 (30.6%)
Medications		
Lipid lowering agents	27,138 (54.7%)	6797 (54.8%)
ACE inhibitors	12,552 (25.3%)	3200 (25.8%)
Beta blockers	17,563 (35.4%)	4304 (34.7%)
Oral hypoglycemics	7343 (14.8%)	1861 (15.0%)
Chronic Conditions		
Hypertension	37,927 (76.5%)	9507 (76.7%)
Hyperlipidemia	35,420 (71.4%)	8872 (71.5%)
Diabetes mellitus	17,736 (35.8%)	4360 (35.2%)
Congestive heart failure	6046 (12.2%)	1430 (11.5%)
Dementia	4616 (9.3%)	1167 (9.4%)
VA utilization		
Emergency department visits		
0	43,986 (88.7%)	10,982 (88.6%)
1	3134 (6.3%)	787 (6.4%)
> 1	2492 (5.0%)	633 (5.1%)
Hospitalizations		
0	47,174 (95.1%)	11,758 (94.8%)
1	1794 (3.6%)	473 (3.8%)
> 1	644 (1.3%)	171 (1.4%)
Functional scores, median (IQR)		
ADL score (range, 0-5)	0 (0-0)	0 (0-0)
IADL score (range, 0-7)	0 (0-1)	0 (0-1)
Medicare linkage available	20,058 (40.4%)	5045 (40.7%)
Medicare utilization		
Emergency department visits		
0	39,643 (79.9%)	9938 (80.1%)
1	5870 (11.8%)	1504 (12.1%)
> 1	4099 (8.3%)	960 (7.7%)
Hospitalizations		
0	44,236 (89.2%)	11,120 (89.7%)
1	3749 (7.6%)	899 (7.2%)
> 1	1627 (3.3%)	393 (3.2%)
Mortality at follow-up		
Year 1	3969 (8.0%)	993 (8.0%)
Year 2	8137 (16.4%)	2021 (16.3%)
Year 3	12,503 (25.2%)	3101 (25.0%)
Year 4	16,720 (33.7%)	4155 (33.5%)
Year 5	20,738 (41.8%)	5185 (41.8%)

*Abbreviations*: *ACE* Angiotensin converting enzyme, *ADL* Activities of daily living, *BMI* Body mass index, *IADL* Instrumental activities of daily living, *IQR* Interquartile range, *SBP* Systolic blood pressure, *SD* Standard deviation, *VA* Veterans Affairs

Roughly 40% of our cohort had Medicare data available (*n* = 20,058 (40.43%) in the development cohort and *n* = 5045 (40.68%) in the validation cohort). The distribution of specific Medicare variables is shown in Supplementary Table S2. For example, related to healthcare utilization, ~ 20% had at least 1 ED visit and ~ 11% had at least 1 hospitalization. When Medicare diagnosis codes were added to the existing VA diagnosis codes, the percentage of individuals with certain diagnoses increased slightly (Supplementary Table S3). For example, in the development cohort, 8.9% had diagnosis codes for congestive heart failure using VA data only which increased to 12.2% using VA and Medicare data.

**Fig. 1:**
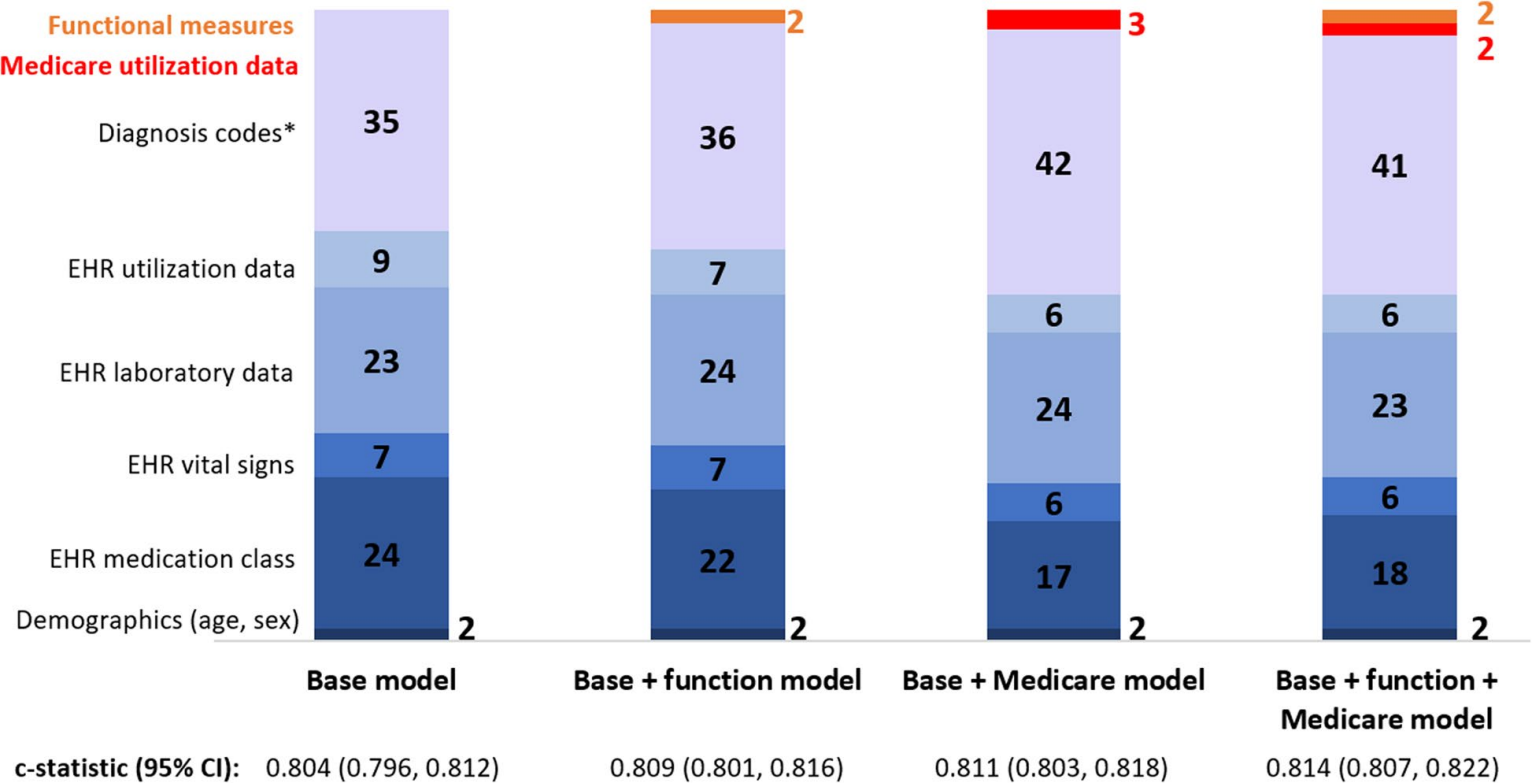
Number of predictor variables selected within each of the 100 variable models. Abbreviations: c-statistic, concordance statistic; EHR, electronic health record. * For the *base* model and *base + function* model, diagnoses codes were obtained only from Veterans Affairs electronic health record data. For the *base + Medicare* and *base + function + Medicare* models, diagnosis codes were obtained from the Veterans Affairs electronic health record and Medicare sources

The 100-predictor *base* model included 2 demographic predictors (age/gender), 35 diagnosis codes, 24 medications, 23 laboratory values, 7 vital sign values, and 9 EHR-derived healthcare utilization predictors (Fig. 1, Supplementary Table S4 and S5). ADL and IADL scores were selected in the final *base + function* and *base + function + Medicare* models. Three Medicare utilization variables were selected in the *base + Medicare* model, and 2 Medicare utilization variables were selected in the *base + function + Medicare* model. Adding Medicare diagnosis codes resulted in more diagnosis code variables being included in models, with the number of diagnosis codes selected increasing from 35 in the *base* model to 42 in the *base + Medicare* model and 41 in the *base + function + Medicare* model.

The c-statistic of the *base* model was 0.809 (95% CI 0.805-0.812) in the development cohort and 0.804 (95% CI 0.796-0.812) in the validation cohort. Adding functional variables and/or Medicare data resulted in slightly higher c-statistics (Table 2). For example, the validation cohort c-statistics of the *base + function, base + Medicare, and base + function + Medicare* models were 0.809 (95% CI 0.801-0.816), 0.811 (95% CI 0.803-0.818), and 0.814 (95% CI 0.807-0.822), respectively.

**Table 2:** Concordance statistics for the four models in the development and validation cohorts

Model	c-statistic (95% CI)	
	Development cohort	Validation cohort
*Base* model	0.809 (0.805, 0.812)	0.804 (0.796, 0.812)
*Base + function* model	0.814 (0.810, 0.818)	0.809 (0.801, 0.816)
*Base + Medicare* model	0.813 (0.810, 0.817)	0.811 (0.803, 0.818)
*Base + function + Medicare* model	0.818 (0.814, 0.822)	0.814 (0.807, 0.822)

*Abbreviations*: *c-statistic* Concordance statistic

The reclassification table comparing the *base* model with the *base + function + Medicare* model is shown in Table 3. Using a threshold of 50%, the NRI for death was 0.0028, meaning that 28 out of 10,000 persons were correctly reclassified by the *base + function + Medicare* model as having died. Similarly, the NRI for not dying was 0.0131, meaning that 131 out of 10,000 persons were correctly reclassified by the *base + function + Medicare* model as surviving to end of follow-up. The overall summary NRI (0.0028 + 0.0131) was 0.0159 (*p* < 0.001). The overall IDI was 0.0176 (*p* < 0.001). Values for the NRI and IDI comparing the *base* model with *base + function* and *base + Medicare* models were similar (Supplementary Table S6). Adding functional measures, Medicare data, or both resulted in small improvements in the fraction of new information provided, ranging from 0.028 to 0.058 (Supplementary Table S7). Calibration plots for all four models suggested close agreement between the observed and predicted probability of mortality across the full risk spectrum (Supplementary Figs. S1-4).

**Table 3:** Reclassification table comparing the *base* model with the *base + function + Medicare* model

	Reclassification table for individuals who did and did not die on follow-up			
Individuals who died during the follow-up period	*Base* model	*Base + function + Medicare* model		
		< 0.5	≥0.5	Total
	< 0.5	6492	**1106**	7598
	≥0.5	*1038*	15,650	16,688
	Total	7530	16,756	24,286
	NRI for events (death)^a^		1106 – 1038 / 24,286 = 0.28%	
Individuals alive at the end of follow-up	< 0.5	18,959	*766*	19,725
	≥0.5	**1099**	4502	5601
	Total	20,058	5268	25,326
	NRI for non-events (survived)^b^		1099 – 766 / 25,326 = 1.31%	
	Overall net reclassification improvement^c^		0.0028 + 0.0131 = 0.0159	
	Integrated discrimination improvement		0.0176	

A risk threshold of 50% was used to calculate the net reclassification improvement for the *base* model compared with the base plus functional measures plus Medicare data model (*base + function + Medicare* model).

*Abbreviations*: *NRI* Net reclassification improvement

^a^The improvement in classification among individuals who died during follow-up is defined as the number of deaths correctly reclassified as higher risk (in boldface = 1106) minus the number of deaths incorrectly reclassified as lower risk (in italics = 1038) divided by the total number of deaths (24,286). Therefore, the NRI for events was 1106 – 1038 / 24,286 = 0.28%

^b^The improvement in classification among individuals who survived during follow-up is defined as the number of non-events correctly reclassified as lower risk (in boldface = 1099) minus the number of non-events incorrectly reclassified as higher risk (in italics = 766) divided by the total number of people who survived (25,326). Therefore, the NRI for non-events was 1099 – 766 / 25,326 = 1.31%

^c^Overall NRI of 0.0159 is the sum of the net percentages of patients that were correctly reclassified (into higher or lower risk depending on whether they subsequently did or did not die) by the model that incorporated function and Medicare data (*base + function + Medicare* model) compared to the *base model*

## Discussion

Incorporating functional measures (ADL and IADL scores) and Medicare data (Medicare utilization variables and diagnosis codes) to a VA EHR-based mortality index involving veterans aged 75 years and older enrolled in primary care clinics resulted in slight improvements in the c-statistic, reclassification indices, and fraction of new information provided. However, the improvements in these measures were small and of questionable clinical relevance. These findings have important implications for deciding which predictor variables to include in EHR-based prognostic models. Documenting functional status with ADL/IADL scores imposes additional time burdens for staff and is not uniformly assessed during healthcare visits. Similarly, additional resources are required to link Medicare data to the existing VA EHR. The small improvements in model performance seen in this study with these data elements may not be worth the added time and effort to include within EHR-based models unless already incorporated for other purposes.

Previously published non-EHR based mortality indices in community-dwelling older adults commonly include a measure of functional status [3, 11]. Functional impairments are clearly important for identifying high risk populations and are associated with numerous adverse health outcomes, including mortality, hospital admissions, nursing home placement, caregiver burnout, and decreased quality of life [9, 25–27]. However, non-EHR based mortality indices typically only include a small number of variables compared with the potentially hundreds of variables in EHR-based indices. Thus, our results, where the addition of functional measures improved model performance only slightly, may be because most of the predictive power of functional measures was already captured with the many other EHR data variables included in the model. For example, healthcare utilization variables in the EHR such as home and community assessments, enrollment in home based primary care, or visits to the social worker may help to identify an individual with functional impairment at higher risk for mortality. In contrast, functional measures may improve model performance in non-EHR models since there are fewer alternative variables whose predictive power substantially overlaps with functional measures.

Despite including only individuals aged 75 years and older, our cohort was relatively free of ADL and IADL dependencies (median of 0 with 92.9 and 70.2% of patients reporting no ADL and IADL dependencies, respectively). Our finding that functional status as measured by reported ADL/IADL dependencies did not significantly improve mortality prediction accuracy therefore may be most relevant when targeting a general primary care population with longer life expectancies. This is aligned with the primary goal of our originally developed life expectancy calculator which was to aid in discussions about longer term clinical decisions, such as cancer screening [8].

While Medicare data did not dramatically increase model performance measures, it did alter the composition of predictors included in the final models. The inclusion of a greater number of diagnosis codes in the *base + function + Medicare* model compared with the *base* model (41 vs. 35, respectively) suggests that Medicare diagnosis codes may add predictive value to the diagnosis codes domain. For example, a patient hospitalized at a non-VA hospital for a medical condition that predicts high mortality, such as septic shock, may now be included within this diagnosis code class through the Medicare diagnosis code. Enriching the VA diagnosis codes with Medicare diagnosis codes allows more diagnosis codes to be included in the final model. However, since the models incorporating Medicare data perform similarly to models without Medicare data, our results suggest that Medicare data may not be necessary for accurate VA EHR mortality prediction models. This may be due to the fact that when EHR models include nearly 100 predictor variables, VA EHR data elements may be able to (indirectly) account for risk factors from Medicare-funded healthcare. For example, even if veterans receive most of their care outside the VA, some comorbidities that are strongly predictive of mortality, such as cancer, may still be entered into the VA EHR during a yearly primary care visit. Similarly, veterans who receive most of their care outside of the VA may access the VA for medications or labs which would allow our model to identify the veteran as higher risk.

In addition to the data linkage burdens associated with adding Medicare data to the existing VA EHR, another issue with using Medicare data is that it is not available in real time. There is typically a 1- to 2-year lag period before the information is available to researchers due to the data processing requirements. Given the small incremental value of adding Medicare data shown in this study, it is likely not worth waiting for this additional data when up-to-date information is already available in the EHR.

Our study has a few important limitations. First, our results speak most directly to VA EHR mortality prediction models in older adults. The VA patient population is known to have important differences in sociodemographic factors, health status, and medical resource use compared to the general patient population [28]. Similarly, the individuals enrolled in the specific VA primary care clinics that collected information on functional status used in this study may not necessarily be representative of the wider national VA cohort. Additional studies in non-VA populations, within different EHR environments, and focused on different clinical outcomes are needed before we can confidently extrapolate our findings to non-VA EHR prediction models. Second, the functional status assessment based on reported ADL/IADL impairments collected during these healthcare visits may not be reflective of an individual’s true severity of functional impairment. However, previous studies have identified VA functional status data as having moderate agreement with a reference standard assessment from trained research assistants [12].

## Conclusions

Overall, we found that incorporating functional measures and Medicare data to an EHR-based mortality prediction model among VA primary care patients aged 75 years and older did not substantially improve model performance measures. This finding is important given that collecting information on functional status and linking Medicare data may be burdensome. While our findings should be replicated in settings outside the VA, researchers developing EHR-based prediction models in large populations with many predictor variables may be able to forego including functional measures and Medicare data with minimal losses in predictive accuracy.

## Supplementary Information

**Additional file 1.**

## Data Availability

The datasets analyzed during the current study are not publicly available since the Veterans Affairs electronic health record data includes protected health information. We are currently exploring with Veterans Affairs data stewards how to de-identify the data so that the de-identified data can be made available from the corresponding author on reasonable request.

## Abbreviations

ADL: Activities of daily living
BIC: Bayesian information criterion
CCS: Clinical Classifications Software
CMS: Centers for Medicare and Medicaid Services
ED: Emergency department
EHR: Electronic health record
HCUP: Healthcare Cost and Utilization Project
IDI: Integrated discrimination improvement
IADL: Instrumental activities of daily living
IAUC: Integrated area under the receiver operating characteristic curve
LASSO: Least absolute shrinkage and selection operator
NRI: Net reclassification improvement
VA: Veterans Affairs
