# Supplementary material for: Do functional status and Medicare claims data improve the predictive accuracy of an electronic health record mortality index? Findings from a national Veterans Affairs cohort
Source: BMC Geriatr. 2022 May 18;22:434. doi: 10.1186/s12877-022-03126-z (PMC9118715; doi:10.1186/s12877-022-03126-z)
Supplement: Supplementary file 1 — Additional file 1. [file 12877_2022_3126_MOESM1_ESM.docx]

**Additional File 1**

**Supplementary Methods**: Additional details regarding methods.

**Supplementary Table S1:** Distribution of activities of daily living and instrumental activities of daily living scores.

**Supplementary Table S2**: Distribution of Medicare variables

**Supplementary Table S3**: Frequency of specific diagnoses using Veterans Affairs diagnosis codes only and after adding Medicare diagnosis codes

**Supplementary Table S4:** Number of predictor variables selected within each model from each of the candidate predictor domains.

**Supplementary Table S5:** List of included predictor variables and associated coefficients in the four final models

**Supplementary Table S6**: Comparisons of the net reclassification improvement and integrated discrimination improvement values across the different models.

**Supplementary Table S7:** Fraction of new information provided for the four models.

**Supplementary Figure S1**: Calibration plot for the *base* model in the validation cohort.

**Supplementary Figure S2**: Calibration plot for the *base+function* model in the validation cohort.

**Supplementary Figure S3**: Calibration plot for the *base+Medicare* model in the validation cohort.

**Supplementary Figure S4**: Calibration plot for the *base+function+Medicare* model in the validation cohort.

**Supplementary References**

**Supplementary Methods:** Additional details regarding methods

**Candidate Variables**

In total, we obtained 854 predictors from the electronic health record (EHR) used for the *base* model. Demographic variables, including age and gender, were extracted from the Clinical Data Warehouse. We created 271 VA specific disease diagnosis variables by collecting International Classification of Diseases, Ninth Revision (ICD-9) diagnosis codes from inpatient and outpatient VA data files and classifying them using the Healthcare Cost and Utilization Project (HCUP) Clinical Classifications Software (CCS) [1]. We identified 365 medications from the Pharmacy Benefits Management file and classified use as any or none in the past year. For the 88 laboratory tests, we created a five-level variable: normal, abnormal low, abnormal high, nonsensical values, or not measured. We extracted data on 9 vital signs from the inpatient and outpatient VA data files, including pulse, temperature, systolic blood pressure, respiration, pain level, body mass index (BMI), weight range, weight change, and pulse oximetry. When multiple options were available for laboratory tests or vital signs, the measurement closest to the index date was chosen. For VA healthcare utilization data, we created 119 types of healthcare visits and categorized visit frequency as zero, one, or at least two visits. These included hospitalizations, emergency department visits, and outpatient visits such as cardiology appointments. For missing data, we performed single stochastic mean imputation using a regression equation with all variables with any missingness included. Variables with missingness were pulse (n=561; 0.9%), temperature (n=3,852; 6.2%), respiration (n=3,251; 5.2%), weight range (n=17,811; 28.7%), weight change (n=17,941; 28.9%), systolic blood pressure (n=545; 0.9%), and body mass index (n=25,330; 40.8%).

**Statistical Analysis**

We assessed the incremental value of the additional predictors (functional measures and Medicare data) to the *base* model in several ways. We compared the concordance statistic (c-statistic) across the four models, which assesses a model’s ability to separate individuals who did and did not have the event of interest. This is equal to the area under the receiver operating characteristic curve. To assess for overfitting, we used the 20% validation cohort (n=12,402) to re-calculate the c-statistic.

We calculated the “fraction of new information provided” to quantify the proportion of variation explained by the additional predictors when added to the *base* model [2]. We first calculated the “fraction of explained risk variation” for each of the four models. This value refers to the R^2^ measure for a binary outcome variable, which expresses the proportion of explained variance by the model [3, 4]. We then calculated the “relative explained variation” by dividing the “fraction of explained risk variation” in each of the three models with additional predictors by the “fraction of explained risk variation” in the *base* model. In other words, the “relative explained variation” refers to the ratio of the variances of predicted values between a model with additional predictors and the *base* model. The “fraction of new information provided” is then calculated as one minus the “relative explained variation.” This refers to the proportion of new information provided by the additional predictors when added to the *base* model.

We calculated reclassification measures such as the net reclassification improvement (NRI) and the integrated discrimination improvement (IDI) [5–7]. NRI measures the degree to which the additional measures were able to appropriately reclassify individuals who did and did not die. More valuable measures tend to increase the predicted mortality risk for individuals who died and decrease the predicted risk for individuals who did not die during the follow-up period. We pre-specified a two-category index with cut-off at 0.50 as we felt that this would represent a meaningful threshold for thinking about average life expectancy. The NRI is calculated as ([number of events reclassified higher – number of events reclassified lower] / total number of events) + ([number of non-events reclassified lower – number of non-events reclassified higher] / total number of non-events). In our case, events are classified as deaths during the follow-up period, and non-events are classified as participants who survived until the end of the follow-up period. The IDI reflects the difference in discrimination slopes between the *base* model and the model including the additional measures. It serves to quantify the value of added measures by calculating improvements in sensitivity and specificity integrated over all possible cut-offs. It is calculated as follows: IDI= (IS_new_ − IS_old_) − (IP_new_ − IP_old_) where IS is the integral of sensitivity over all possible cut-off values from the (0,1) interval, IP is the integral of “one minus specificity” over all possible cut-off values from the (0,1 interval), new refers to the model including the additional predictors, and old refers to the *base* model. Calibration, which refers to the agreement between observed outcomes and predictors, was assessed visually by plotting the predicted probability of mortality (x-axis) by the observed proportion of mortality (y-axis) [8]. All statistical analyses were conducted using SAS version 9.4 (SAS Institute, Inc) and R version 4.03 (R Project for Statistical Computing).

**Supplementary Table S1:** Distribution of activities of daily living and instrumental activities of daily living scores

|  | **Development cohort (n=49,612)** | | **Validation cohort (n=12,402)** | |
| --- | --- | --- | --- | --- |
|  | **Frequency** | **Percent (%)** | **Frequency** | **Percent (%)** |
| **Activities of daily living score** | | | | |
| 0 | 46,105 | 92.93 | 11,559 | 93.20 |
| 1 | 1,399 | 2.82 | 357 | 2.88 |
| 2 | 733 | 1.48 | 153 | 1.23 |
| 3 | 380 | 0.77 | 82 | 0.66 |
| 4 | 653 | 1.32 | 153 | 1.23 |
| 5 | 342 | 0.69 | 98 | 0.79 |
| **Instrumental activities of daily living score** | | | | |
| 0 | 34,829 | 70.2 | 8,644 | 69.70 |
| 1 | 4,281 | 8.63 | 1,065 | 8.59 |
| 2 | 2,976 | 6.00 | 805 | 6.49 |
| 3 | 2,102 | 4.24 | 531 | 4.28 |
| 4 | 1,762 | 3.55 | 470 | 3.79 |
| 5 | 1,378 | 2.78 | 316 | 2.55 |
| 6 | 1,373 | 2.77 | 354 | 2.85 |
| 7 | 911 | 1.84 | 217 | 1.75 |

**Supplementary Table S2:** Distribution of Medicare variables

|  | **Development cohort (N=49,612)** | | **Validation cohort (N=12,402)** | |
| --- | --- | --- | --- | --- |
|  | **Frequency** | **Percent (%)** | **Frequency** | **Percent (%)** |
| **Medicare linkage available** | | | | |
|  | 20,058 | 40.43 | 5,045 | 40.68 |
| **Length of stay at skilled nursing facility (days)** | | | | |
| 0 | 47,878 | 96.5 | 11,947 | 96.33 |
| 1-10 | 406 | 0.82 | 107 | 0.86 |
| 11-20 | 470 | 0.95 | 119 | 0.96 |
| 21-30 | 272 | 0.55 | 72 | 0.58 |
| 30+ | 586 | 1.18 | 157 | 1.27 |
| **Days of home health services** | | | | |
| 0 | 47,643 | 96.03 | 11,942 | 96.29 |
| 1-20 | 491 | 0.99 | 121 | 0.98 |
| 21-40 | 586 | 1.18 | 134 | 1.08 |
| 41-60 | 500 | 1.01 | 107 | 0.86 |
| 60+ | 392 | 0.79 | 98 | 0.79 |
| **Number of durable medical equipment** | | | | |
| 0 | 42,451 | 85.57 | 10,648 | 85.86 |
| 1 | 2,350 | 4.74 | 609 | 4.91 |
| 2-3 | 1,696 | 3.42 | 390 | 3.14 |
| 4-8 | 1,487 | 3 | 374 | 3.02 |
| 9+ | 1,628 | 3.28 | 381 | 3.07 |
| **Emergency department visits** | | | | |
| 0 | 39,643 | 79.91 | 9,938 | 80.13 |
| 1 | 5,870 | 11.83 | 1,504 | 12.13 |
| 2+ | 4,099 | 8.26 | 960 | 7.74 |
| **Hospitalizations** | | | | |
| 0 | 44,236 | 89.16 | 11,120 | 89.66 |
| 1 | 3,749 | 7.56 | 899 | 7.17 |
| 2+ | 1,627 | 3.28 | 393 | 3.17 |
| **Outpatient visits** | | | | |
| 0 | 28,985 | 58.42 | 7,227 | 58.27 |
| 1 | 3,868 | 7.8 | 951 | 7.67 |
| 2+ | 16,759 | 33.78 | 4,224 | 34.06 |

**Supplementary Table S3:** Frequency of specific diagnoses using Veterans Affairs diagnosis codes only and after adding Medicare diagnosis codes

|  | **Development cohort (N=49,612)** | | **Validation cohort (N=12,402)** | |
| --- | --- | --- | --- | --- |
| **Veterans Affairs diagnosis codes only** | | | | |
|  | **Frequency** | **Percent (%)** | **Frequency** | **Percent (%)** |
| Hypertension | 35,817 | 72.19 | 8,970 | 72.33 |
| Diabetes without complication | 16,379 | 33.01 | 4,039 | 32.57 |
| Diabetes with complication | 4,123 | 8.31 | 957 | 7.72 |
| Hyperlipidemia | 33,719 | 67.97 | 8,447 | 68.11 |
| Chronic Kidney Disease | 6,525 | 13.15 | 1,620 | 13.06 |
| Congestive heart failure | 4,422 | 8.91 | 1,061 | 8.56 |
| Dementia | 4,086 | 8.24 | 1,009 | 8.14 |
| COPD | 8,662 | 17.46 | 2,121 | 17.1 |
| **Veterans Affairs and Medicare diagnosis codes** | | | | |
|  | **Frequency** | **Percent (%)** | **Frequency** | **Percent (%)** |
| Hypertension | 37,927 | 76.45 | 9,507 | 76.66 |
| Diabetes without complication | 17,736 | 35.75 | 4,360 | 35.16 |
| Diabetes with complication | 5,358 | 10.82 | 1,312 | 10.58 |
| Hyperlipidemia | 35,420 | 71.39 | 8,872 | 71.54 |
| Chronic Kidney Disease | 8,151 | 16.43 | 2,003 | 16.15 |
| Congestive heart failure | 6,046 | 12.19 | 1,430 | 11.53 |
| Dementia | 4,616 | 9.3 | 1,167 | 9.41 |
| COPD | 10,167 | 20.49 | 2,482 | 20.01 |

Abbreviations: COPD, chronic obstructive pulmonary disease

**Supplementary Table S4**: Number of predictor variables selected within each model from each of the candidate predictor domains

| **Predictor domain** | **Number of potential variables** | **Base model** | **Base + function model** | **Base + Medicare model** | **Base + function + Medicare model** |
| --- | --- | --- | --- | --- | --- |
| Demographics (age, gender) | 2 | 2 | 2 | 2 | 2 |
| EHR medication class | 365 | 24 | 22 | 17 | 18 |
| EHR vital signs | 9 | 7 | 7 | 6 | 6 |
| EHR laboratory data | 88 | 23 | 24 | 24 | 23 |
| EHR utilization data | 119 | 9 | 7 | 6 | 6 |
| Diagnosis codes* | 277 | 35 | 36 | 42 | 41 |
| Functional measures (ADL & IADL scores) | 2 | . | 2 | . | 2 |
| Medicare utilization data | 6 | . | . | 3 | 2 |
| Total variables | 868 | 100 | 100 | 100 | 100 |

Abbreviations: ADL, activities of daily living; EHR, electronic health record; IADL, instrumental activities of daily living; VA, Veterans Affairs

* For the *base* model and *base+function* model, diagnoses codes were obtained only from Veterans Affairs electronic health record data. For the *base+Medicare* and *base+function+Medicare* models, diagnosis codes were obtained from the Veterans Affairs electronic health record and Medicare sources. The 277 diagnosis codes indicate 6 unique diagnosis codes from Medicare data in addition to the 271 overlapping diagnosis codes.

**Supplementary Table S5:** List of included predictor variables and associated coefficients in the four final models

| **Predictor variable** | **Label** | **Base model** | **Base + function model** | **Base + Medicare model** | **Base + function + Medicare model** |
| --- | --- | --- | --- | --- | --- |
| **Demographics** |  |  |  |  |  |
| Age | cube_age1 | 0.057 | 0.054 | 0.056 | 0.053 |
| age cubic spline1 | cube_age2 | -0.030 | -0.025 | -0.033 | -0.028 |
| age cubic spline2 | cube_age3 | 0.112 | 0.094 | 0.122 | 0.104 |
| Gender | gender | 0.276 | 0.252 | 0.283 | 0.266 |
| **Diagnosis codes** |  |  |  |  |  |
| Immunizations and screening for infectious disease | ccs_10 | -0.045 | -0.039 | -0.061 | -0.057 |
| Coronary atherosclerosis and other heart disease | ccs_101 | 0.099 | 0.101 | 0.081 | 0.083 |
| Nonspecific chest pain | ccs_102 |  |  | -0.141 | -0.133 |
| Other and ill-defined heart disease | ccs_104 |  | 0.083 |  |  |
| Cardiac dysrhythmias | ccs_106 | 0.109 | 0.116 | 0.080 | 0.086 |
| Congestive heart failure; nonhypertensive | ccs_108 | 0.242 | 0.240 | 0.224 | 0.222 |
| Acute cerebrovascular disease | ccs_109 | 0.182 | 0.153 | 0.157 | 0.140 |
| Late effects of cerebrovascular disease | ccs_113 | 0.194 |  | 0.162 |  |
| Peripheral and visceral atherosclerosis | ccs_114 | 0.145 | 0.147 | 0.134 | 0.133 |
| Aortic; peripheral; and visceral artery aneurysms | ccs_115 | 0.110 | 0.107 | 0.084 | 0.085 |
| Other circulatory disease | ccs_117 | 0.052 |  |  |  |
| Chronic obstructive pulmonary disease and bronchiectasis | ccs_127 | 0.170 | 0.168 | 0.150 | 0.149 |
| Asthma | ccs_128 |  |  | -0.080 | -0.076 |
| Pleurisy; pneumothorax; pulmonary collapse | ccs_130 | 0.196 | 0.203 |  |  |
| Respiratory failure; insufficiency; arrest (adult) | ccs_131 | 0.230 | 0.214 | 0.142 | 0.147 |
| Other upper respiratory disease | ccs_134 | -0.080 | -0.062 | -0.083 | -0.074 |
| Esophageal disorders | ccs_138 | -0.040 | -0.040 | -0.052 | -0.049 |
| Cancer of liver and intrahepatic bile duct | ccs_16 | 1.096 | 1.136 | 0.941 | 0.963 |
| Calculus of urinary tract | ccs_160 |  |  |  | -0.103 |
| Hyperplasia of prostate | ccs_164 | -0.071 | -0.071 | -0.071 | -0.067 |
| Cancer of bronchus; lung | ccs_19 | 0.419 | 0.425 | 0.359 | 0.370 |
| Chronic ulcer of skin | ccs_199 | 0.219 | 0.190 | 0.179 | 0.154 |
| Rheumatoid arthritis and related disease | ccs_202 | 0.171 | 0.156 | 0.131 | 0.121 |
| Osteoarthritis | ccs_203 | -0.052 | -0.052 | -0.049 | -0.045 |
| Other non-traumatic joint disorders | ccs_204 |  |  | -0.042 | -0.040 |
| Osteoporosis | ccs_206 | 0.136 | 0.127 | 0.139 | 0.134 |
| Other connective tissue disease | ccs_211 |  | -0.049 | -0.042 | -0.043 |
| Intracranial injury | ccs_233 |  |  |  | -0.231 |
| Other injuries and conditions due to external causes | ccs_244 | 0.132 | 0.116 | 0.091 | 0.072 |
| Abdominal pain | ccs_251 |  |  | -0.084 | -0.074 |
| Other screening for suspected conditions (not mental disorders or infectious disease) | ccs_258 |  |  | -0.048 | -0.042 |
| E Codes: Fall | ccs_2603 |  |  |  | 0.117 |
| Hodgkin’s disease | ccs_37 |  |  |  | 0.719 |
| Non-Hodgkin’s lymphoma | ccs_38 | 0.195 | 0.203 | 0.161 | 0.159 |
| Leukemias | ccs_39 | 0.167 | 0.172 |  |  |
| Multiple myeloma | ccs_40 | 0.727 | 0.715 | 0.658 | 0.655 |
| Secondary malignancies | ccs_42 | 0.769 | 0.761 | 0.572 | 0.580 |
| Neoplasms of unspecified nature or uncertain behavior | ccs_44 | 0.119 | 0.121 |  |  |
| Maintenance chemotherapy; radiotherapy | ccs_45 |  |  | 0.251 | 0.252 |
| Other and unspecified benign neoplasm | ccs_47 | -0.057 |  | -0.066 | -0.052 |
| Diabetes mellitus without complication | ccs_49 | 0.057 | 0.050 | 0.052 |  |
| Diabetes mellitus with complications | ccs_50 |  |  | 0.067 | 0.058 |
| Nutritional deficiencies | ccs_52 |  | 0.061 |  |  |
| Disorders of lipid metabolism | ccs_53 | -0.062 | -0.057 | -0.079 | -0.067 |
| Deficiency and other anemia | ccs_59 | 0.057 | 0.051 | 0.071 | 0.069 |
| Anxiety disorders | ccs_651 |  | -0.063 |  |  |
| Delirium, dementia, and amnestic and other cognitive disorders | ccs_653 | 0.334 | 0.230 | 0.328 | 0.228 |
| Schizophrenia and other psychotic disorders | ccs_659 |  |  | 0.200 | 0.167 |
| Screening and history of mental health and substance abuse codes | ccs_663 | 0.124 | 0.124 | 0.065 | 0.070 |
| Parkinson`s disease | ccs_79 | 0.446 | 0.391 | 0.410 | 0.348 |
| Other eye disorders | ccs_91 |  |  | -0.051 | -0.042 |
| Other ear and sense organ disorders | ccs_94 | -0.058 | -0.054 | -0.065 | -0.060 |
| **Veterans Affairs Medication Class** |  |  |  |  |  |
| ANTIHISTAMINES,OTHER | ah109 |  | -0.076 |  |  |
| ANTINEOPLASTIC, OTHER | an900 | 0.309 | 0.305 | 0.275 | 0.275 |
| BLOOD FORMATION PRODUCTS | bl400 | 0.416 | 0.429 | 0.415 | 0.427 |
| OPIOID ANALGESICS | cn101 | 0.066 | 0.061 | 0.074 | 0.070 |
| ANTIDEPRESSANTS,OTHER | cn609 | 0.078 | 0.067 | 0.077 |  |
| ANTIPSYCHOTICS,OTHER | cn709 | 0.169 |  |  |  |
| CNS MEDICATIONS,OTHER | cn900 | 0.257 | 0.219 | 0.250 | 0.209 |
| DIGITALIS GLYCOSIDES | cv050 | 0.120 | 0.115 |  |  |
| BETA BLOCKERS/RELATED | cv100 | 0.040 | 0.038 | 0.047 | 0.043 |
| ANTILIPEMIC AGENTS | cv350 | -0.050 | -0.046 | -0.042 | -0.044 |
| LOOP DIURETICS | cv702 | 0.150 | 0.140 | 0.142 | 0.131 |
| GENITO-URINARY AGENTS,OTHER | gu900 | -0.124 | -0.107 | -0.126 | -0.109 |
| INSULIN | hs501 | 0.130 | 0.116 | 0.104 | 0.100 |
| ORAL HYPOGLYCEMIC AGENTS,ORAL | hs502 | 0.052 | 0.052 |  | 0.067 |
| IMMUNE SUPPRESSANTS | im600 | 0.337 | 0.344 | 0.301 | 0.309 |
| NONSALICYLATE NSAIs,ANTIRHEUMATIC | ms102 | -0.058 |  |  |  |
| BRONCHODILATORS,ANTICHOLINERGIC | re105 | 0.151 | 0.152 | 0.149 | 0.150 |
| ANTIASTHMA,OTHER | re109 | 0.071 | 0.073 | 0.066 | 0.079 |
| RESPIRATORY AGENTS,OTHER | re900 | 0.574 | 0.531 | 0.580 | 0.557 |
| ENTERAL NUTRITION | tn200 | 0.238 | 0.217 | 0.230 | 0.204 |
| PROSTHETICS/SUPPLIES/DEVICES | xa000 |  | -1.523 |  |  |
| DIAPERS | xa305 | 0.201 |  | 0.210 |  |
| PADS/DIAPERS,OTHER | xa399 | 0.244 | 0.165 | 0.240 | 0.163 |
| PROTECTANTS,SKIN,OSTOMY | xa604 | 0.273 | 0.253 | 0.269 | 0.249 |
| SYRINGES/NEEDLES,OTHER | xa859 | -0.539 | -0.554 |  |  |
| **Vital Signs** |  |  |  |  |  |
| **Body Mass Index Category** |  |  |  |  |  |
| 18.5-25 | bmi_cat_2 | -0.014 | 0.004 | -0.036 | -0.019 |
| 25-30 | bmi_cat_3 | -0.084 | -0.063 | -0.107 | -0.088 |
| 30-35 | bmi_cat_4 | -0.111 | -0.094 | -0.139 | -0.121 |
| >35 | bmi_cat_5 | -0.080 | -0.069 | -0.111 | -0.100 |
|  |  |  |  |  |  |
| **Pulse** | pls | 0.004 | 0.004 | 0.004 | 0.004 |
| **Pulse Oximetry Category** |  |  |  |  |  |
| 93-95 | po_cat_2 | -0.209 | -0.204 | -0.199 | -0.195 |
| >96 or missing | po_cat_3 | -0.222 | -0.215 | -0.213 | -0.209 |
| **Respiration** | rp | 0.011 | 0.010 | 0.010 | 0.010 |
| **Systolic Blood Pressure category** |  |  |  |  |  |
| 90-104 | sys_cat_2 | -0.084 | -0.087 | -0.091 | -0.082 |
| 105-119 | sys_cat_3 | -0.190 | -0.184 | -0.192 | -0.176 |
| 120-139 | sys_cat_4 | -0.211 | -0.201 | -0.213 | -0.192 |
| 140-159 | sys_cat_5 | -0.201 | -0.187 | -0.199 | -0.177 |
| 160-179 | sys_cat_6 | -0.157 | -0.142 | -0.150 | -0.129 |
| >180 | sys_cat_7 | -0.130 | -0.117 | -0.133 | -0.115 |
| **Weight Range** | weight_range | 0.003 | 0.003 | 0.003 | 0.003 |
| **Weight Change** | wt_chg | -0.002 | -0.003 |  |  |
| **Laboratory data** |  |  |  |  |  |
| **PSA (Prostatic Specific Antigen)** |  |  |  |  |  |
| abnormal high (>4ng/ml) | lab_flag_19_1 | 0.037 | 0.039 | 0.035 | 0.035 |
| Nonsensical value | lab_flag_19_98 | -0.086 | -0.074 | -0.089 | -0.077 |
| Not Done | lab_flag_19_99 | 0.053 | 0.047 | 0.057 | 0.049 |
| **Hemoglobin** |  |  |  |  |  |
| abnormal High (M >17.5g/dL \|\|F>15.3g/DL) | lab_flag_1_1 | 0.011 | -0.002 | 0.118 | 0.110 |
| abnormal Low(M<14g/DL \|\| F<12.3g/DL) | lab_flag_1_2 | 0.154 | 0.154 | 0.142 | 0.137 |
| Nonsensical value | lab_flag_1_98 | 0.346 | 0.346 | 0.514 | 0.476 |
| Not Done | lab_flag_1_99 | 0.489 | 0.480 | 0.013 | 0.007 |
| **Folic Acid/Folate** |  |  |  |  |  |
| abnormal High(>17ng/mL) | lab_flag_25_1 | -0.062 | -0.056 | -0.062 | -0.057 |
| abnormal Low (<2.7ng/mL) | lab_flag_25_2 | 0.000 |  | 0.000 | 0.000 |
| Nonsensical value | lab_flag_25_98 | -0.063 | -0.051 | -0.065 | -0.056 |
| Not Done | lab_flag_25_99 | -0.100 | -0.100 | -0.102 | -0.102 |
| Triglycerides |  |  |  |  |  |
| abnormal High(>150mg/dL) | lab_flag_30_1 | -0.008 | -0.018 | -0.007 | -0.016 |
| Nonsensical value | lab_flag_30_98 | 0.168 | 0.041 | 0.244 | 0.183 |
| Not Done | lab_flag_30_99 | 0.065 | 0.054 | 0.067 | 0.057 |
| **Serum Creatinine** |  |  |  |  |  |
| abnormal High (M>1.3mg/dL\|\|F>1.2mg/dL) | lab_flag_31_1 | 0.112 | 0.109 | 0.104 | 0.097 |
| abnormal Low (M<0.8 mg/dL \|\|F<0.6 mg/dL) | lab_flag_31_2 | 0.059 | 0.050 | 0.058 | 0.089 |
| Nonsensical value | lab_flag_31_98 | -0.199 | -0.123 | -0.151 | -0.042 |
| Not Done | lab_flag_31_99 | -0.029 | -0.046 | -0.008 | -0.006 |
| **Sodium (Serum)** |  |  |  |  |  |
| abnormal High(>145 mEq/L) | lab_flag_3_1 | 0.082 | 0.075 |  |  |
| abnormal Low (<136 mEq/L) | lab_flag_3_2 | 0.056 | 0.058 |  |  |
| Not Done | lab_flag_3_99 | 0.112 | 0.111 |  |  |
| **ALT (Alanine Aminotransferase)** |  |  |  |  |  |
| Abnormal High (M>40 U/L \|\| F>19 U/L) | lab_flag_45_1 | -0.080 | -0.080 | -0.076 | -0.075 |
| Abnormal Low (M<10 U/L \|\|F<4 U/L) | lab_flag_45_2 | 0.228 | 0.216 | 0.227 | 0.215 |
| Nonsensical value | lab_flag_45_98 | 0.218 | 0.210 | 0.225 | 0.215 |
| Not Done | lab_flag_45_99 | 0.016 | 0.019 | 0.028 | 0.035 |
| **Alkaline Phosphatase (Serum)** |  |  |  |  |  |
| Abnormal High (>147 IU/L) | lab_flag_48_1 | 0.336 | 0.336 | 0.313 | 0.314 |
| Abnormal Low (<44 IU/L) | lab_flag_48_2 | -0.074 | -0.069 | -0.074 | -0.073 |
| Nonsensical value | lab_flag_48_98 | 0.082 | 0.060 | 0.086 | 0.103 |
| Not Done | lab_flag_48_99 | 0.031 | 0.023 | 0.019 | 0.019 |
| **Albumin (Serum)** |  |  |  |  |  |
| abnormal High (>5.4 g/dL) | lab_flag_49_2 | 0.295 | 0.275 | 0.290 | 0.272 |
| Not Done | lab_flag_49_99 | 0.036 | 0.037 | 0.032 | 0.033 |
| **Hematocrit** |  |  |  |  |  |
| Abnormal High (M>50.4% \|\| F>44.6%) | lab_flag_50_1 | 0.118 | 0.121 |  |  |
| Abnormal Low (M<41.5% \|\| F<36.9%) | lab_flag_50_2 | 0.007 | 0.003 |  |  |
| Nonsensical value | lab_flag_50_98 | 0.000 |  |  |  |
| Not Done | lab_flag_50_99 | -0.481 | -0.489 |  |  |
| **Microalbumin/Creatinine Ratio** |  |  |  |  |  |
| Abnormal High (>30 mg/g) | lab_flag_56_1 | 0.146 | 0.147 | 0.144 | 0.144 |
| Nonsensical value | lab_flag_56_98 | -0.016 | -0.013 | -0.025 | -0.017 |
| Not Done | lab_flag_56_99 | 0.079 | 0.073 | 0.072 | 0.064 |
| **Troponin I** |  |  |  |  |  |
| Abnormal High (>0.05 ng/mL) | lab_flag_59_1 | 0.156 | 0.139 | 0.159 | 0.148 |
| Nonsensical value | lab_flag_59_98 | -0.124 | -0.126 | -0.125 | -0.126 |
| Not Done | lab_flag_59_99 | -0.023 | -0.031 | -0.061 | -0.059 |
| **BUN (Blood Urea Nitrogen)** |  |  |  |  |  |
| Abnormal High (>23mg/dL) | lab_flag_5_1 | 0.090 | 0.091 | 0.084 | 0.085 |
| Abnormal Low (<8 mg/dL) | lab_flag_5_2 | -0.097 | -0.119 | -0.112 | -0.111 |
| Nonsensical value | lab_flag_5_98 | 0.000 | 0.000 |  | 0.000 |
| Not Done | lab_flag_5_99 | -0.027 | -0.028 | -0.002 | -0.001 |
| **Bilirubin (Direct)** |  |  |  |  |  |
| Abnormal High (0.3 mg/dL) | lab_flag_60_1 | 0.115 | 0.115 | 0.111 | 0.116 |
| Nonsensical value | lab_flag_60_98 | 0.048 | 0.060 | 0.031 | 0.039 |
| Not Done | lab_flag_60_99 | 0.000 | -0.003 | -0.003 | -0.007 |
| **Carbon Dioxide** |  |  |  |  |  |
| Abnormal High (>29 mEq/L) | lab_flag_64_1 | 0.029 | 0.028 | 0.025 | 0.025 |
| Abnormal Low (<23 mEq/L) | lab_flag_64_2 | 0.118 | 0.113 | 0.117 | 0.114 |
| Nonsensical value | lab_flag_64_98 | -0.379 | -0.461 | -0.178 | -0.246 |
| Not Done | lab_flag_64_99 | 0.123 | 0.117 | 0.135 | 0.130 |
| **Chloride (Serum)** |  |  |  |  |  |
| Abnormal High (>107 mEq/L) | lab_flag_65_1 | -0.032 | -0.029 | -0.024 | -0.020 |
| Abnormal Low (<98 mEq/L) | lab_flag_65_2 | 0.210 | 0.198 | 0.217 | 0.207 |
| Not Done | lab_flag_65_99 | -0.122 | -0.121 | -0.094 | -0.080 |
| **estimated Glomerular Filtration Rate (eGFR) based on creatinine** |  |  |  |  |  |
| Abnormal High (>120 mL/min/1.73m2 ) | lab_flag_66_1 | 0.162 | 0.140 | 0.159 | -0.152 |
| Abnormal Low (<90 mL/min/1.73m2) | lab_flag_66_2 | -0.028 | -0.030 | -0.028 | 0.092 |
| Nonsensical value | lab_flag_66_98 | 0.033 | 0.012 | 0.030 | 0.267 |
| Not Done | lab_flag_66_99 | 0.016 | 0.029 | 0.010 | -0.120 |
| **Partial pressure of oxygen (PO2)** |  |  |  |  |  |
| Abnormal High (>100 mmHg ) | lab_flag_69_1 | -0.132 | -0.143 | -0.153 |  |
| Abnormal Low (<70 mmHg) | lab_flag_69_2 | 0.109 | 0.096 | 0.108 |  |
| Nonsensical value | lab_flag_69_98 | 0.386 | 0.331 | 0.379 |  |
| Not Done | lab_flag_69_99 | -0.086 | -0.095 | -0.113 |  |
| **Total white blood cell count)** |  |  |  |  |  |
| Abnormal High (>10 K/uL ) | lab_flag_6_1 | 0.193 | 0.184 | 0.205 | 0.196 |
| Abnormal Low (<5 K/uL) | lab_flag_6_2 | -0.082 | -0.070 | -0.089 | -0.077 |
| Nonsensical value | lab_flag_6_98 | 0.003 | 0.019 | 0.059 | 0.073 |
| Not Done | lab_flag_6_99 | -0.044 | -0.027 | -0.082 | -0.084 |
| **Total Protein (Serum)** |  |  |  |  |  |
| Abnormal High (>8.3g/dL ) | lab_flag_71_1 | 0.226 | 0.222 | 0.232 | 0.230 |
| Abnormal Low (<6.0 g/dL) | lab_flag_71_2 | 0.068 | 0.077 | 0.065 | 0.070 |
| Nonsensical value | lab_flag_71_98 | -0.421 | -0.472 | -0.426 | -0.485 |
| Not Done | lab_flag_71_99 | -0.034 | -0.025 | -0.031 | -0.024 |
| **Pro B Natriuretic Peptide** |  |  |  |  |  |
| Abnormal High | lab_flag_74_1 | 0.255 | 0.252 | 0.255 | 0.245 |
| Abnormal Low | lab_flag_74_2 | 0.332 | 0.348 | 0.430 | 0.402 |
| Not Done | lab_flag_74_99 | 0.209 | 0.204 | 0.187 | 0.172 |
| **Platelet Count** |  |  |  |  |  |
| Abnormal High (>400 k/mm3) | lab_flag_77_1 | 0.142 | 0.146 | 0.133 | 0.131 |
| Abnormal Low (<150 k/mm3) | lab_flag_77_2 | 0.090 | 0.088 | 0.087 | 0.084 |
| Nonsensical | lab_flag_77_98 | 0.243 | 0.240 | 0.206 | 0.192 |
| Not Done | lab_flag_77_99 | 0.108 | 0.112 | 0.133 | 0.143 |
| **Digoxin** |  |  |  |  |  |
| Abnormal High (>0.8 NG/ML) | lab_flag_7_1 |  |  | 0.114 | 0.093 |
| Abnormal Low (<0.5 NG/ML) | lab_flag_7_2 |  |  | -0.010 | -0.022 |
| Nonsensical | lab_flag_7_98 |  |  | 0.249 | 0.230 |
| Not Done | lab_flag_7_99 |  |  | -0.053 | -0.063 |
| **AST (Aspartate Aminotransferase)** |  |  |  |  |  |
| Abnormal High (>41 U/L) | lab_flag_9_1 | 0.103 | 0.094 | 0.102 | 0.101 |
| Abnormal Low (<10 U/L) | lab_flag_9_2 | 0.112 | 0.103 | 0.109 | 0.102 |
| Nonsensical | lab_flag_9_98 | 0.204 | 0.224 | 0.181 | 0.201 |
| Not Done | lab_flag_9_99 | -0.029 | -0.033 | -0.024 | -0.029 |
| **Veterans Affairs Utilization Data** |  |  |  |  |  |
| **Blind Rehabilitation Outpatient Specialist** |  |  |  |  |  |
| 1 | util_ao_1 | 0.000 |  | 0.000 |  |
| 1+ | util_ao_2 | 0.233 |  | 0.230 |  |
| **Dermatology** |  |  |  |  |  |
| 1 | util_au_1 | -0.310 | -0.313 |  |  |
| 1+ | util_au_2 | -0.097 | -0.090 |  |  |
| **Primary care/medicine** |  |  |  |  |  |
| 1 | util_bm_1 |  |  | 0.016 | 0.016 |
| 1+ | util_bm_2 |  |  | 0.059 | 0.060 |
| **Cardiac stress test** |  |  |  |  |  |
| 1 | util_bt_1 | 0.000 | 0.000 |  |  |
| 1+ | util_bt_2 | -0.242 | -0.223 |  |  |
| **Optometry** |  |  |  |  |  |
| 1 | util_cg_1 | -0.068 | -0.077 |  |  |
| 1+ | util_cg_2 | -0.048 | -0.040 |  |  |
| **Anesthesia pre/post-operative consult** |  |  |  |  |  |
| 1+ | util_cp_2 | -0.153 | -0.156 | -0.133 | -0.126 |
| **Cystoscopy room unit** |  |  |  |  |  |
| 1 | util_cw_1 |  | 0.000 |  | 0.000 |
| 1+ | util_cw_2 |  | 0.138 | 0.136 | 0.149 |
| **Home/Community Assessment** |  |  |  |  |  |
| 1 | util_en_1 | 0.400 |  |  |  |
| 1+ | util_en_2 | 0.224 |  |  |  |
| **Inpatient Visit** |  |  |  |  |  |
| 1 | util_inpatient_1 | -0.251 | -0.231 | -0.200 | -0.190 |
| 1+ | util_inpatient_2 | -0.432 | -0.374 | -0.343 | -0.313 |
| **Social Work Service** |  |  |  |  |  |
| 1 | util_o_1 | -0.108 | -0.199 | -0.071 | -0.164 |
| 1+ | util_o_2 | 0.143 | 0.113 | 0.139 | 0.105 |
| **Computed Tomography (CT)** |  |  |  |  |  |
| 1 | util_r_1 |  |  | 0.106 | 0.109 |
| 1+ | util_r_2 |  |  | 0.063 | 0.072 |
| **Home-based primary care (HBPC)** |  |  |  |  |  |
| 1+ | util_x_2 | 0.119 |  | 0.162 |  |
| **Dental** |  |  |  |  |  |
| 1 | util_z_1 | -0.842 | -0.815 | -0.804 | -0.772 |
| 1+ | util_z_2 | -0.115 | -0.112 | -0.115 | -0.120 |
| **Functional Status** |  |  |  |  |  |
| sum of Activities of Daily Living (ADL) score | adl_total |  | 0.080 |  | 0.079 |
| sum of Instrumental Activities of Daily Living (IADL) score | iadl_total |  | 0.077 |  | 0.075 |
| **Medicare Utilization Data** |  |  |  |  |  |
| **Medicare durable medical equipment (DME)** |  |  |  |  |  |
| 1 | cms_dme_1 |  |  | 0.017 | 0.024 |
| 2-3 | cms_dme_2 |  |  | 0.004 | 0.011 |
| 4-8 | cms_dme_3 |  |  | 0.017 | 0.024 |
| 9+ | cms_dme_4 |  |  | 0.162 | 0.164 |
| **Medicare emergency department visit** |  |  |  |  |  |
| 1 | cms_er_1 |  |  | 0.063 | 0.066 |
| 1+ | cms_er_2 |  |  | 0.107 | 0.114 |
| **Days of home health services** |  |  |  |  |  |
| 1-20 | cms_hh_1 |  |  | 0.088 |  |
| 21-40 | cms_hh_2 |  |  | 0.119 |  |
| 41-60 | cms_hh_3 |  |  | 0.098 |  |
| 60+ | cms_hh_4 |  |  | 0.227 |  |

**Supplementary Table S6**: Comparisons of the net reclassification improvement and integrated discrimination improvement values across the different models.

|  | **Categorical NRI at 50% threshold** | **P-value** | **IDI** | **P-value** |
| --- | --- | --- | --- | --- |
| *Base* model vs.  *base+function* model | 0.0076 | <0.001 | 0.0104 | <0.001 |
| *Base* model vs.  b*ase+Medicare* model | 0.0099 | <0.001 | 0.0083 | <0.001 |
| *Base* model vs.  *base+function+Medicare* model | 0.0159 | <0.001 | 0.0176 | <0.001 |

Abbreviations: IDI, integrated discrimination improvement; NRI, net reclassification improvement

**Supplementary Table S7:** Fraction of new information provided for the four models

| **Model** | **Fraction of explained risk variation^a^** | **Relative explained variation^b^** | **Fraction of new information^c^** |  |
| --- | --- | --- | --- | --- |
|  |  |  |  |  |
| *Base* model | 0.286 | . | . |  |
| *Base+function* model | 0.297 | 0.965 | 0.035 |  |
| *Base+Medicare* model | 0.294 | 0.972 | 0.028 |  |
| *Base+function+Medicare* model | 0.304 | 0.942 | 0.058 |  |

^a^ Fraction of explained risk variation refers to the R^2^ measure for a binary outcome variable.

^b^ Relative explained variation is the ratio of the “fraction of explained risk variation” in the *base* model to the “fraction of explained risk variation” in the model with additional predictors. For example, the *base* model explains 96.5% of the variation explained by the *base+function* model.

^c^ Fraction of new information is calculated as one minus the relative explained variation. It refers to the fraction of new information provided by the additional predictors when added to the *base* model.

**Supplementary Figure S1**: Calibration plot for the *base* model in the validation cohort


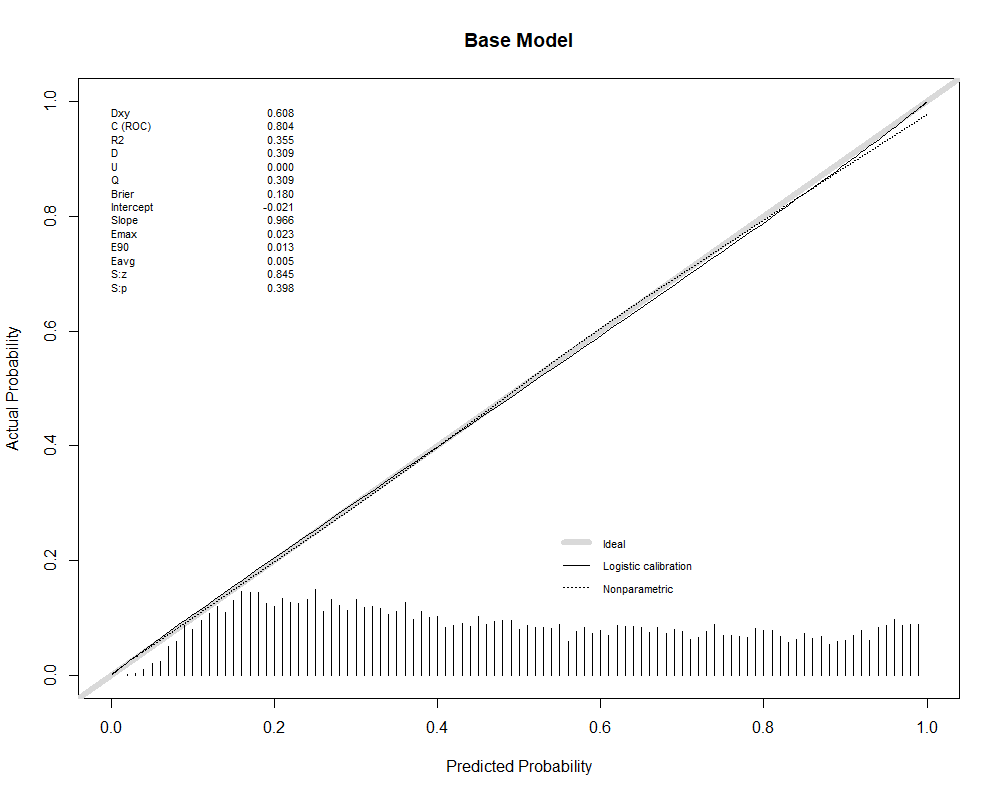


Note. A histogram of predicted probabilities is placed above the x-axis.

**Supplementary Figure S2:** Calibration plot for the *base+function* model in the validation cohort.


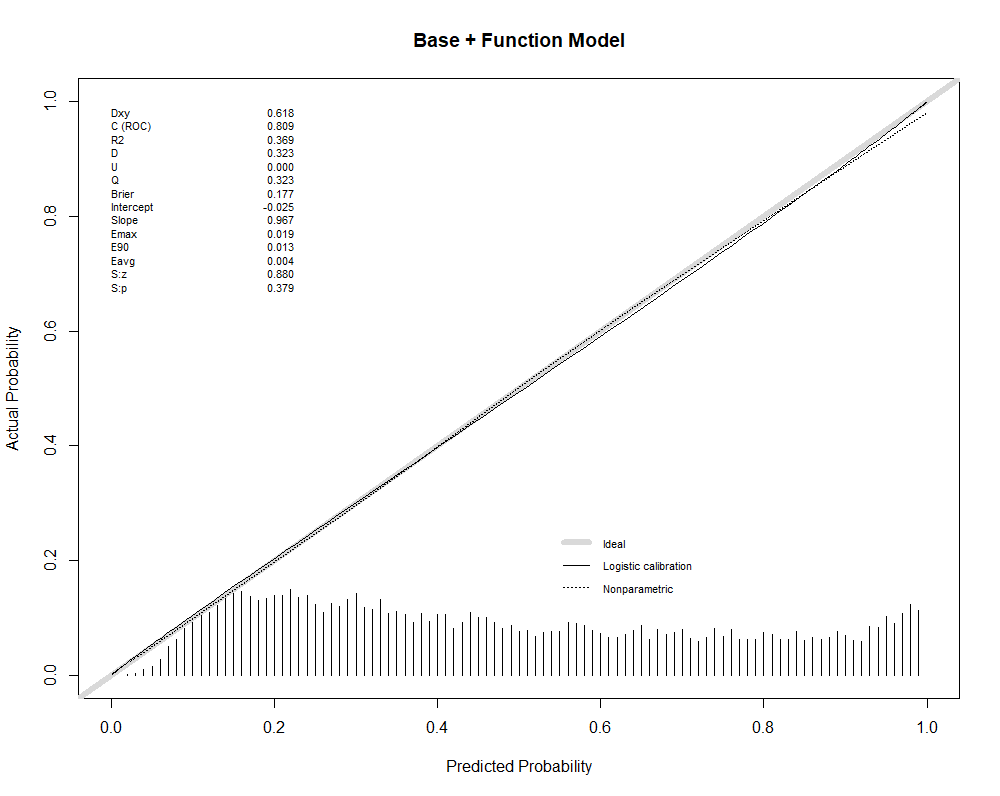


Note. A histogram of predicted probabilities is placed above the x-axis.

**Supplementary Figure S3:** Calibration plot for the *base+Medicare* model in the validation cohort


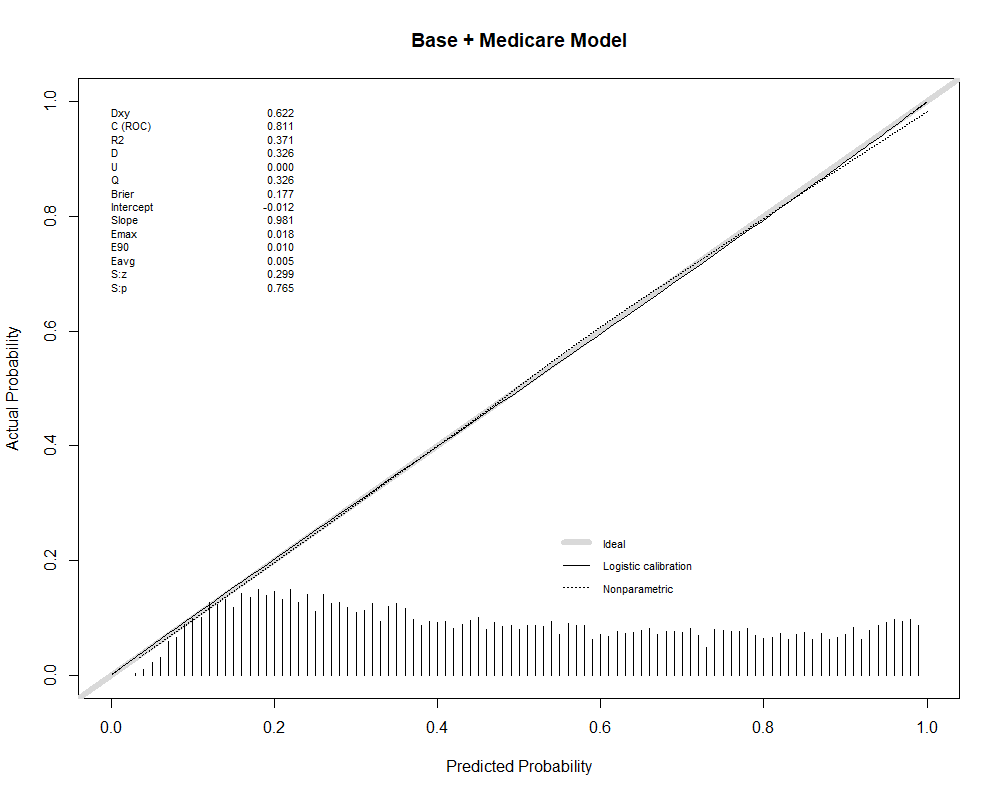


Note. A histogram of predicted probabilities is placed above the x-axis.

**Supplementary Figure S4:** Calibration plot for the *base+function+Medicare* model in the validation cohort


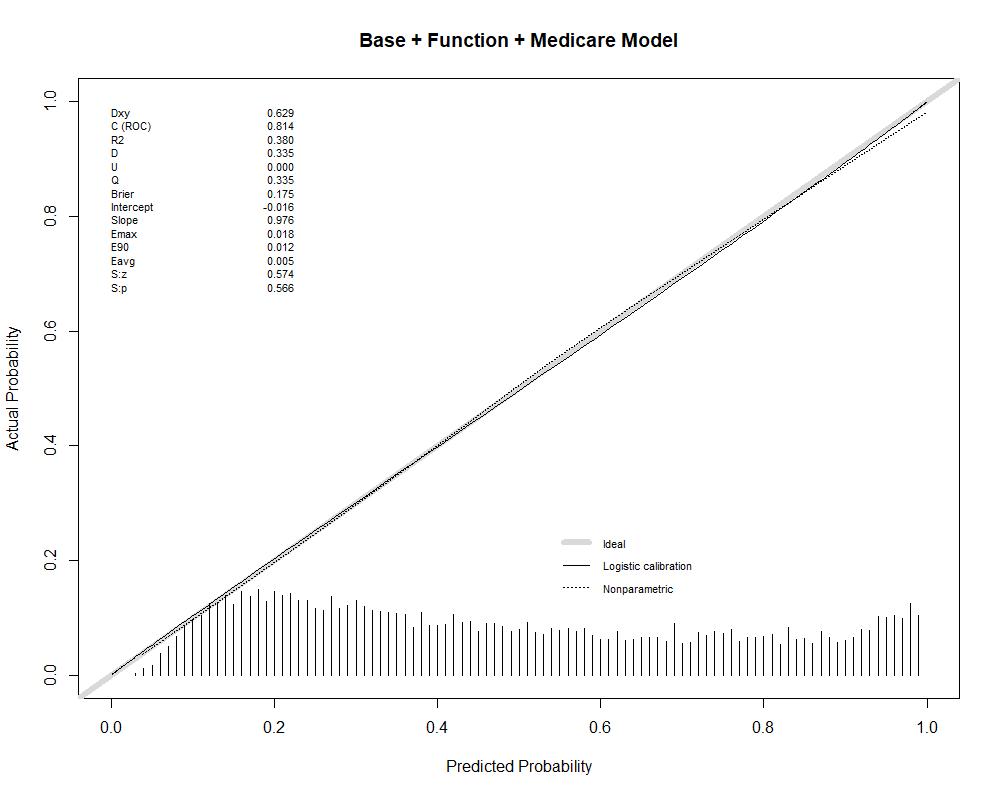


Note. A histogram of predicted probabilities is placed above the x-axis.

**Supplementary References**

1. Clinical Classifications Software (CCS) for ICD-9-CM. https://www.hcup-us.ahrq.gov/toolssoftware/ccs/ccs.jsp. Accessed 14 Jul 2021.

2. Harrell F. Statistically efficient ways to quantify added predictive value of new measurements. 2020. https://www.fharrell.com/post/addvalue/. Accessed 14 Jul 2021.

3. Mittlböck M, Schemper M. Explained variation for logistic regression. Stat Med. 1996;15:1987–97.

4. Schemper M, Henderson R. Predictive accuracy and explained variation in Cox regression. Biometrics. 2000;56:249–55.

5. Pencina MJ, D’Agostino RB, D’Agostino RB, Vasan RS. Evaluating the added predictive ability of a new marker: From area under the ROC curve to reclassification and beyond. Stat Med. 2008;27:157–72; discussion 207-212.

6. Leening MJG, Vedder MM, Witteman JCM, Pencina MJ, Steyerberg EW. Net reclassification improvement: computation, interpretation, and controversies: A literature review and clinician’s guide. Ann Intern Med. 2014;160:122–31.

7. Kerr KF, Wang Z, Janes H, McClelland RL, Psaty BM, Pepe MS. Net reclassification indices for evaluating risk-prediction instruments: A critical review. Epidemiol Camb Mass. 2014;25:114–21.

8. Steyerberg EW, Vickers AJ, Cook NR, Gerds T, Gonen M, Obuchowski N, et al. Assessing the performance of prediction models: A framework for some traditional and novel measures. Epidemiol Camb Mass. 2010;21:128–38.
